# Identification and validation of glycosaminoglycan metabolism-associated immune biomarkers in patients with ischemic stroke based on weighted gene co-expression network analysis and machine learning

**DOI:** 10.3389/fneur.2026.1852526

**Published:** 2026-07-28

**Authors:** Bing Xie, Yangsong Ou, Qiuhong Liu, Yuan Li

**Affiliations:** Department of Neurology, The First Hospital of Changsha, Affiliated Changsha Hospital of Xiangya School of Medicine, Central South University, Changsha, China

**Keywords:** biomarkers, glycosaminoglycan metabolism, immune infiltration, ischemic stroke, machine learning

## Abstract

**Background:**

Acute ischemic stroke (IS) represents a cerebrovascular emergency characterized by abrupt cessation of cerebral perfusion and subsequent systemic immune activation. Accumulating evidence suggests that glycosaminoglycan (GAG) metabolic pathways critically modulate neuroinflammatory cascades and vascular dysfunction. However, the role of GAG metabolism in generating peripheral molecular signatures and translatable diagnostic indicators remains poorly characterized. This investigation sought to identify GAG metabolism–associated immune biomarkers and decode their immunometabolic mechanisms in IS.

**Methods:**

We integrated two transcriptomic repositories (GSE58294 and GSE16561) and compiled 127 GAG metabolism–related genes from published literature. Weighted gene co-expression network analysis was used to extract GAG-associated modules. Machine-learning algorithms combined with differential expression validation prioritized candidate biomarkers, and diagnostic performance was assessed via nomogram construction. Mechanistic insights were obtained through gene set enrichment analysis and computational immune deconvolution, while pharmacological databases identified potential therapeutic compounds. Biomarker expression was experimentally validated using RT-qPCR in peripheral blood samples obtained from 5 IS patients (3 males and 2 females; mean age 67.6 years, range 55–77 years) and 5 healthy controls (2 males and 3 females; mean age 68.6 years, range 40–88 years). Blood samples from IS patients were collected 3–7 days after stroke onset, whereas control samples were obtained on the second day after hospital admission.

**Results:**

Three biomarkers, MCEMP1, PRRG4, and VSIG4, demonstrated concordant transcriptional elevation in IS samples. The constructed nomogram exhibited robust discriminatory accuracy, confirming its clinical utility. Pathway enrichment revealed associations with ribosomal biogenesis, neutrophil extracellular trap formation, and neuroactive ligand–receptor signaling, indicating roles in translational regulation and innate immunity. Immune profiling disclosed substantial alterations in neutrophils, M0 macrophages, CD8^+^ T cells, and eosinophils, with strong correlations between biomarkers and neutrophil-driven inflammation. Drug–gene interaction analysis identified trichostatin A as a shared molecular target. RT-qPCR confirmed a significant upregulation of MCEMP1 and PRRG4.

**Conclusion:**

MCEMP1, PRRG4, and VSIG4 constitute reliable molecular indicators that link systemic GAG metabolic dysregulation to IS-associated immune reorganization. These findings advance our mechanistic understanding of GAG-mediated inflammatory networks in stroke and establish actionable targets for both clinical risk stratification and therapeutic development.

## 1 Introduction

Ischemic stroke (IS) is a leading cause of mortality and disability among neurological disorders, characterized by acute cerebral arterial occlusion that induces local hypoperfusion and energy crisis. This subsequently triggers a cascade of reactions, such as excitotoxicity and mitochondrial damage, ultimately leading to neuronal necrosis and glial responses (1, 2). Despite growing understanding of ischemia–reperfusion–associated “neuro-vascular-immune” crosstalk, IS remains difficult to treat owing to its narrow therapeutic window and marked individual heterogeneity. According to the latest Global Burden of Disease 2021 estimates, stroke, including IS, remains a major global health burden. It contributes substantially to mortality and disability-adjusted life years, particularly in low- and middle-income countries, and is a key driver of the overall burden of neurological diseases (3, 4). Currently available clinical treatments, such as intravenous thrombolysis and mechanical thrombectomy, can markedly improve vascular recanalization rates. However, the frequent occurrence of “futile recanalization” due to constraints such as a narrow time window and high imaging screening thresholds indicates that simple vascular recanalization does not fully translate to clinical functional benefits. Adjuvant therapeutic strategies such as neuroprotection and immune regulation thus remain unmet clinical needs (5–7).

Against this backdrop, glycosaminoglycans (GAGs) and their associated proteoglycans, as core components of the endothelial glycocalyx and cerebral extracellular matrix (ECM), have been identified as a key “glycobiological axis” that regulates vascular homeostasis, immune adhesion, and blood–brain barrier (BBB) permeability (8). Ischemia–reperfusion injury can induce rapid shedding and remodeling of the endothelial glycocalyx, accompanied by alterations in the structure and charge density of GAGs such as heparan sulfate. This process further exacerbates leukocyte adhesion, microcirculatory “no-reflow” phenomenon, and BBB leakage, ultimately amplifying neuroinflammatory responses and cerebral tissue damage (9). Furthermore, GAGs and their proteoglycans play dual functions as a ligand library and a signaling “scaffold” in the “immune-synapse-regeneration” regulatory network of the central nervous system, modulating key pathways such as complement activation and neuroplasticity. These findings suggest that GAGs are not merely passive damaged structures but rather regulatable core nodes in IS pathology (10). While these studies have elucidated the pathological mechanisms of GAG metabolism in brain tissue, the molecular characteristics of GAG alterations in peripheral blood and their value as clinically applicable biomarkers remain unclear (11, 12). Most existing research has focused on the fundamental role of the cerebral endothelial glycocalyx in maintaining BBB function, but the systemic reflection of acute-phase “GAG metabolic imbalance-immune microenvironment remodeling” in peripheral blood and its coupled regulatory mechanisms remain insufficiently clarified.

To address these research limitations, this study leveraged publicly available transcriptomic cohort datasets to comprehensively delineate the IS molecular landscape, with emphasis on GAG metabolism–related signaling networks. Genes exhibiting strong associations with GAG metabolic processes were prioritized through differential gene expression profiling, weighted gene co-expression network analysis (WGCNA), and pathway enrichment strategies. Reliable biomarker candidates were subsequently identified by integrating multiple machine-learning algorithms with expression-level validation, culminating in the development of a clinically applicable nomogram for predictive modeling and performance assessment. Through the synergistic application of computational bioinformatics, machine-learning frameworks, and regulatory network dissection methodologies, this research endeavors to provide systematic molecular evidence regarding GAG metabolism in IS pathophysiology. Ultimately, this work aspires to establish a robust theoretical foundation, facilitate the discovery of disease-specific biomarkers, and unveil potential therapeutic targets for IS management.

## 2 Materials and methods

### 2.1 Data collection

Two IS-related transcriptomic datasets were obtained from the Gene Expression Omnibus (GEO; https://www.ncbi.nlm.nih.gov/geo/). GSE58294 (platform: GPL570), comprising peripheral whole blood from 69 IS patients and 23 healthy controls, served as the training dataset. GSE16561 (platform: GPL6883), containing blood samples from 39 IS patients and 24 controls, was used as an independent external validation dataset. For the training dataset (GSE58294), raw CEL files were processed with the RMA algorithm (R package affy) for background correction, quantile normalization, and log_2_ transformation. Probe IDs were mapped to gene symbols via the hgu133plus2.db package, with multi-probe aggregation using the mean for genes with multiple probes, and protein-coding genes were retained via biomaRt filtering. For validation set GSE16561, expression values were extracted from the GEO matrix file, with probe-to-gene mapping conducted using the gene symbol field in platform annotations, followed by log_2_ transformation and quantile normalization to retain protein-coding genes. Due to differences in detection principles and dynamic ranges between platforms, independent Z-score normalization was applied to both datasets during cross-dataset comparison to eliminate inter-platform systematic bias (11). Overall, 127 GAG metabolism–related genes were compiled by integrating data from four published studies (12–15) for subsequent analyses (Supplementary Table 1).

### 2.2 Differential expression analysis

Differentially expressed genes (DEGs) between IS patients and controls were identified in the training dataset GSE58294 using the R package limma (v3.56.2) (16), with a threshold of *p* < 0.05 and |log_2_ fold change (FC)| > 1. A volcano plot was generated to visualize the DEG distribution, highlighting the top 10 upregulated and downregulated genes, and a heatmap was constructed to display their expression patterns across groups.

### 2.3 WGCNA

To assess the enrichment of GAG metabolism in IS, the GAG scores for each sample in GSE58294 were calculated using the single-sample gene set enrichment analysis (ssGSEA) algorithm implemented in the R package GSVA (v1.53.28) (17), with group differences assessed by the Wilcoxon test (*p* < 0.05). WGCNA was subsequently applied using the WGCNA package (v1.73) (18) to identify GAG-associated co-expression modules. Outlier samples were identified through hierarchical clustering, and the optimal soft-thresholding power (*β*) was determined using the pickSoftThreshold function based on the scale-free topology criterion (R^2^ > 0.8). The gene adjacency matrix was transformed into a topological overlap matrix (TOM), followed by dynamic tree cutting to delineate gene modules (minimum module size = 350, deepSplit = 4, merging threshold = 0.1). Module–trait relationships were then assessed by Pearson correlation analysis between module eigengenes and GAG scores (|cor| > 0.3, *p* < 0.05), and the most significantly correlated module was designated as the key GAG-associated module.

### 2.4 Enrichment and protein–protein interaction (PPI) network analysis

Candidate GAG-related genes in IS were obtained by overlapping DEGs with genes from the key GAG-associated module. Gene Ontology (GO) and Kyoto Encyclopedia of Genes and Genomes (KEGG) enrichment analyses were conducted using the R package clusterProfiler (v4.15.0.003) (19) (*p* < 0.05), covering three GO categories: biological process (BP), cellular component (CC), and molecular function (MF). A PPI network was constructed by retrieving protein interaction data from the STRING database (https://string-db.org/; interaction score > 0.15) and visualized using Cytoscape (v3.9.1) (20).

### 2.5 Identification of biomarkers

Three machine-learning algorithms were applied to the candidate gene expression profiles to select feature genes. Least absolute shrinkage and selection operator (LASSO) regression was performed using the R package glmnet (v4.1-8) (21) with 5-fold cross-validation, retaining genes with non-zero coefficients at *λ*.1se (alpha = 1, type.measure = “deviance”). Random forest (RF) analysis was implemented via the R package randomForest (v4.7-1.2) (22) with 5-fold cross-validation (ntree = 66, mtry = 11, importance = TRUE), and genes exceeding the mean Gini index were selected. Support vector machine–recursive feature elimination (SVM–RFE) was carried out using the R package e1071 (v1.7-16) (21) with an RBF kernel and 5-fold cross-validation, retaining genes with weights exceeding zero. Feature genes were defined as the intersection of the results from all three algorithms. Differential expression of feature genes was validated in both datasets using the Wilcoxon test, and those with consistent significance (*p* < 0.05) and concordant expression trends were confirmed as biomarkers. The log_2_ FCs of biomarkers in stroke versus control groups were calculated for training (GSE58294) and validation (GSE16561) sets. Merged log_2_FC data were used to generate a scatter plot (training vs. validation log_2_FC, with *y* = *x* and zero lines) to show directional consistency, plus a Bland–Altman plot (mean log_2_FC vs. difference, with ± 1.96 SD limits) to quantify cross-platform differences. Based on the training set (GSE58294), Spearman correlation was performed between biomarkers and core GAG metabolism genes (*HPSE*, *EXT1*, *CHST2*, and *SULF1*). Correlation heatmaps and scatterplot matrices with linear fitting were generated using ggplot2 (v3.5.2) to visualize expression associations.

### 2.6 Nomogram construction and evaluation

A logistic regression nomogram model was constructed based on the biomarkers screened from the training set (GSE58294) using the R package rms (v6.8-1) (23). Internal validation was performed using the bootstrap resampling method (*B* = 1,000 resamples) and calculated the area under the receiver operating characteristic curve (AUC) and calibration slope before and after correction. Model calibration was evaluated using calibration curves and the Hosmer–Lemeshow goodness-of-fit test (*p* > 0.05) via the R package regplot (v1.1) (24). Clinical utility was assessed by decision curve analysis (DCA) using the R package ggDCA (v1.1) (25). Discriminative performance was quantified by receiver operating characteristic (ROC) analysis and AUC calculation using the R package pROC (v1.18.5) (26). External validation was performed by applying the trained model to the validation set (GSE16561) for DCA and ROC analysis.

### 2.7 Gene set enrichment analysis (GSEA)

GSEA was performed to investigate the biological pathways associated with each biomarker using transcriptomic data from GSE58294. Canonical pathway gene sets (C2 CP) were downloaded from the Molecular Signatures Database (c2.cp.v2023.1.Hs.symbols.gmt). For each biomarker, Spearman correlation coefficients with all other genes were calculated using the cor function from the R package stats (v4.3.3) (27), and genes ranked by decreasing correlation served as the GSEA input. Enrichment was conducted via the R package clusterProfiler (v4.15.0.003) (19), with significance defined as |normalized enrichment score (NES)| > 1, false discovery rate (FDR) < 0.25, and *p* < 0.05.

### 2.8 Immune infiltration analysis

Immune cell infiltration was quantified using the CIBERSORT algorithm (v0.1.0) (28) to estimate the relative abundance of 22 immune cell types (29). Samples with *p* > 0.05 or total immune cell proportions outside the range of 0.95–1.05 were excluded. Differentially infiltrating immune cells between the IS and control groups were identified by the Wilcoxon test (*p* < 0.05). Spearman correlation analysis was then performed using the R package psych (v2.4.6.26) (30) to assess associations among differential immune cells and between biomarkers and differential immune cells (|cor| > 0.3, *p* < 0.05). Spearman’s rank correlation analysis was performed to calculate the correlation coefficient (r) and significance level (*p*-value) between each biomarker and the immune cell surface marker genes. Finally, faceted heatmaps were generated using ggplot2 (v3.5.2) to visualize the correlation results, stratified by dataset (training/validation) and cell type (neutrophils, M0 macrophages, CD8^+^ T cells, and eosinophils).

### 2.9 Chromosome and subcellular localization analyses

Chromosomal localization of each biomarker was determined using the R package RCircos (v1.2.2) (31). The subcellular localization of the corresponding encoded proteins was predicted via the GeneCards database^1^ to infer their cellular functional compartments.

### 2.10 GeneMANIA and molecular regulatory network analysis

Functional interaction networks among biomarkers were constructed using GeneMANIA^2^ based on *Homo sapiens*. MicroRNAs (miRNAs) targeting the biomarkers were predicted by integrating results from TargetScan^3^ and miRDB,^4^ with only overlapping miRNAs retained. Regulatory transcription factors (TFs) were retrieved from hTFtarget.^5^ Both TF–mRNA and miRNA–mRNA regulatory networks were visualized using Cytoscape.

### 2.11 Potential therapeutic drug prediction

Drug–gene interaction analysis was performed using the DSigDB^6^ to identify compounds potentially targeting the biomarkers. The resulting biomarker–drug interaction networks were visualized using Cytoscape.

### 2.12 Clinical trial validation of biomarkers

To preliminarily explore the clinical relevance of the identified biomarkers, RT-qPCR was conducted on blood specimens obtained from Changsha First Hospital, comprising 5 IS patients and 5 healthy volunteers (total *n* = 10). The patients’ baseline clinical characteristics are presented in Supplementary Table 1. All subjects provided written informed consent before enrollment, and the protocol received ethical approval from the institutional review board of Changsha First Hospital (approval number: 2024120). RNA was extracted from peripheral blood using TRIzol reagent (R401-01, Vazyme, China) according to the manufacturer’s instructions. Reverse transcription was subsequently conducted using HP All-in-One qRT Master Mix II RT203-Ver.1 (24Y0124; YoungGen, China), with the thermal cycling program executed on an S1000™ Thermal Cycler (Bio-Rad, USA). Table 1 lists all primer sequences used in this study. Amplification reactions were run on a CFX Connect real-time PCR detection system (XLFZ006; Bio-Rad, USA), and transcript abundance was calculated using the 2^−ΔΔCT^ method. Statistical power analysis for the independent samples t-test was performed using G*Power (version 3.1.9.7) (32). Statistical analysis and figure generation were performed in GraphPad Prism 10.^7^

**Table 1:** RT-qPCR primer sequences.

Primer	Sequence
MCEMP1 F	TACAGAGCCATCCTGAGCCT
MCEMP1 R	TCGCATGCTTGTACGGAGTT
PRRG4 F	TCACTCCCGGCAACCTAGAA
PRRG4 R	CGGCTGAAGAGCTGATTTTGTG
VSIG4 F	GGCCAACGACTCTGGAGAAA
VSIG4 R	TTGCCATTGATCTGGGCGAT
GAPDH F	ATGGGCAGCCGTTAGGAAAG
GAPDH R	AGGAAAAGCATCACCCGGAG

### 2.13 Specific analysis of biomarker expression at the subpopulation level in circulating immune cells

Based on RNA expression data from the Human Protein Atlas Blood Atlas database, the expression values (normalized transcripts per million [nTPM]) of the biomarkers were extracted across various circulating immune cell subsets, including neutrophils, monocytes, B cells, T cells, and NK cells. Following data cleaning and normalization, the data were sorted by immune lineage, and heatmaps were generated using ggplot2 (v3.5.2) to illustrate the expression abundance and distribution specificity of the biomarkers among different immune cell subsets (32).

### 2.14 Statistical analysis

All statistical analyses were performed using R software (version 4.2.2). Group differences were assessed using the Wilcoxon rank-sum test, and a two-tailed *p*-value of less than 0.05 was considered statistically significant.

## 3 Results

### 3.1 Identification of 76 DEGs and 1,558 GAG module genes

**Figure 1:**
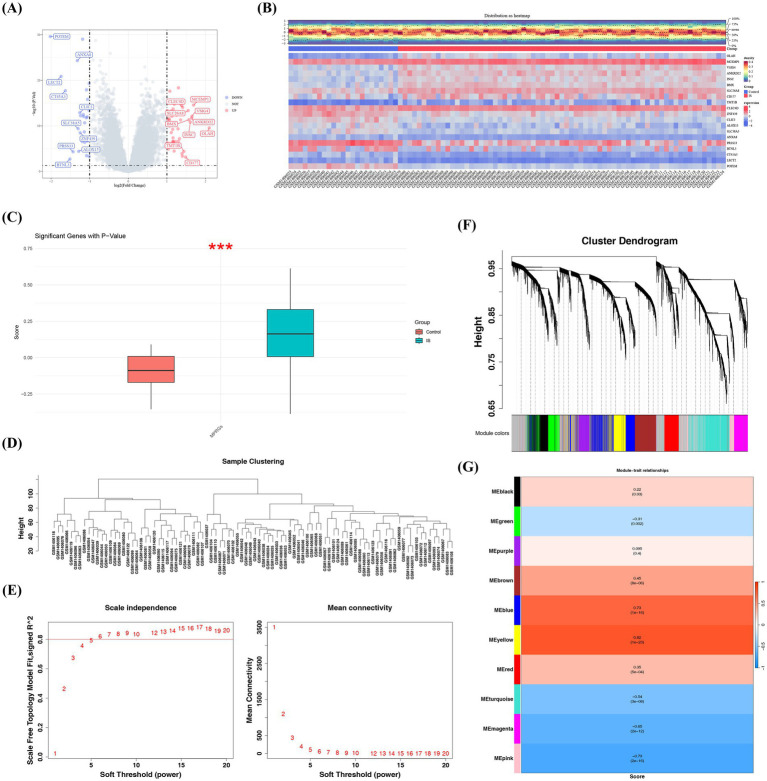
Differential expression and WGCNA identification of glycosaminoglycan metabolism–related modules in ischemic stroke. **(A)** Volcano plot of differentially expressed genes between the IS and control samples. **(B)** Heatmap of representative differentially expressed genes. **(C)** Comparison of glycosaminoglycan metabolism scores between groups. **(D)** Sample clustering dendrogram for outlier detection. **(E)** Analysis of the scale-free topology fit index and mean connectivity for soft-threshold selection. **(F)** Gene dendrogram and module assignment identified by WGCNA. **(G)** Correlation heatmap between module eigengenes and glycosaminoglycan metabolism scores.

Differential expression analysis of the training dataset GSE58294 identified 76 DEGs between IS patients and healthy controls, comprising 46 upregulated and 30 downregulated genes in the IS group (Figures 1A,B). ssGSEA-based scoring demonstrated that GAG scores were significantly elevated in IS relative to controls (*p* < 0.05), indicating a prominent shift in GAG metabolic activity during IS pathophysiology (Figure 1C). This finding provided the rationale for subsequent WGCNA. Hierarchical clustering confirmed the absence of outlier samples (Figure 1D), ensuring data integrity for downstream analyses. A soft-thresholding power of *β* = 6 met the scale-free topology criterion (*R*^2^ = 0.820) and was adopted for network construction (Figure 1E). TOM-based hierarchical clustering delineated 10 co-expression modules, excluding the gray module that represented non-co-expressed genes (Figure 1F). The MEyellow module exhibited the most significant positive association with GAG scores (cor = 0.82, *p* = 1 × 10^−23^) (Figure 1G), and the 1,558 constituent genes were accordingly designated as GAG module genes, representing candidate functional drivers of aberrant GAG metabolism in IS.

### 3.2 Functional characterization of 30 candidate genes

**Figure 2:**
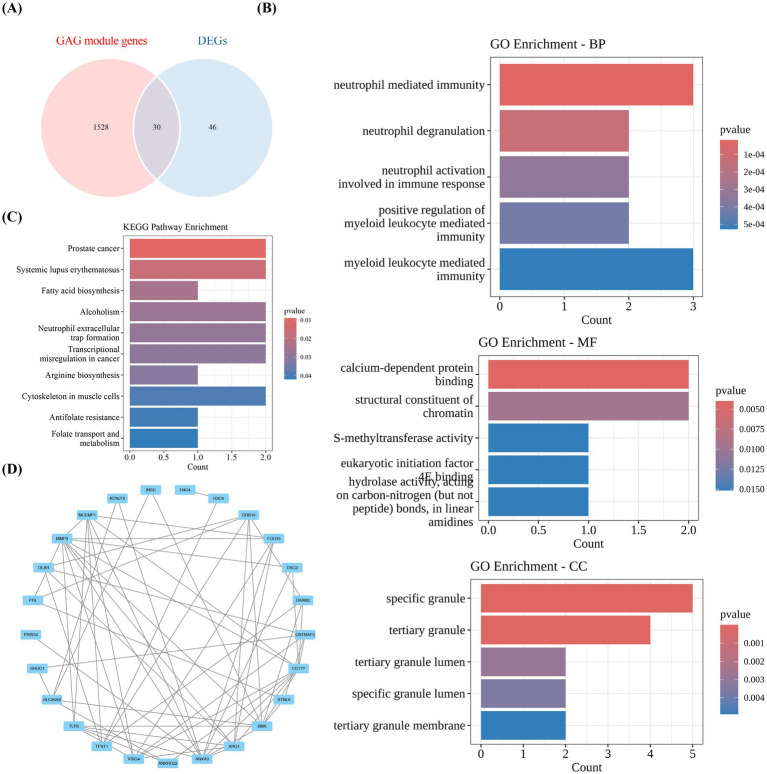
Functional characterization of candidate genes associated with glycosaminoglycan metabolism in ischemic stroke. **(A)** Venn diagram showing overlap between differentially expressed genes and genes from the key WGCNA module. **(B)** GO enrichment analysis of the candidate genes. **(C)** KEGG enrichment analysis of the candidate genes. **(D)** Protein–protein interaction network of candidate genes.

Intersecting the DEGs with GAG module genes yielded 30 candidate genes (Figure 2A). GO enrichment analysis identified 212 significantly enriched terms (*p* < 0.05), encompassing 167 BPs, 16 CCs, and 29 MFs (Figure 2B; Supplementary Table 2). Within the BP category, candidate genes were predominantly associated with immune regulation, neutrophil degranulation, and neutrophil activation involved in the immune response. Enriched CCs included specific granules, tertiary granules, and tertiary granule lumens, while enriched MFs comprised S-methyltransferase activity, hydrolase activity, and calcium-dependent protein binding. KEGG analysis further identified 11 significantly enriched pathways (*p* < 0.05) (Figure 2C; Supplementary Table 2), with representative pathways including fatty acid biosynthesis, neutrophil extracellular trap formation, and the cytoskeleton in muscle cells. These results collectively suggest that neutrophil-mediated immune activation and metabolic–immune coupling may jointly contribute to inflammatory injury in IS. PPI network analysis showed that 25 candidate genes formed 62 interaction pairs (Figure 2D). Notably, *CD177*, *BMX*, and *ANXA3* exhibited extensive connectivity within the network, implying their potential functional roles in disease-related biological regulation.

### 3.3 MCEMP1, PRRG4, and VSIG4 were identified as biomarkers

**Figure 3:**
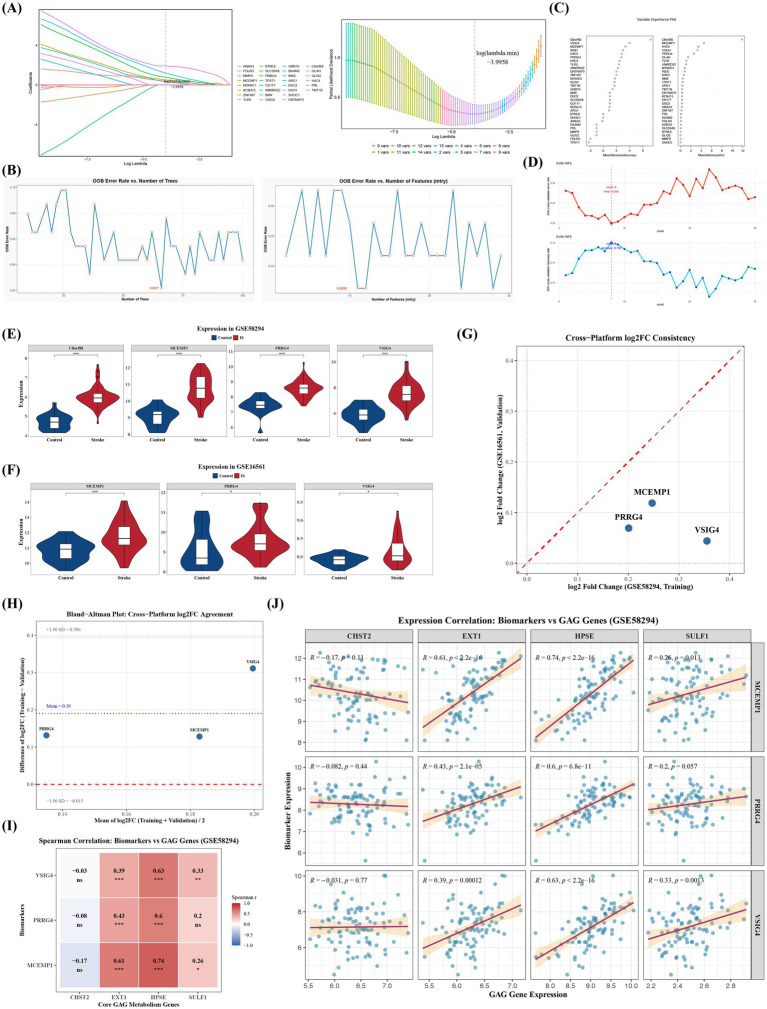
Machine learning–based identification and validation of ischemic stroke biomarkers. **(A)** LASSO coefficient profiles and cross-validation results. **(B, C)** Random forest model construction and feature importance ranking. **(D)** Feature selection by SVM–RFE. **(E, F)** Expression validation of feature genes in the training and validation datasets, ^*^p < 0.05, ^**^p < 0.01, ^***^p < 0.001. **(G)** Cross-platform log_2_FC consistency scatter plot. **(H)** Cross-platform log_2_FC consistency Bland–Altman plot. **(I)** Spearman Correlation of Biomarkers vs. Core GAG Metabolism Genes. **(J)** Scatterplot matrix of expression correlation between biomarkers and core GAG genes.

Using three machine-learning algorithms (LASSO, RF, and SVM–RFE) on 30 candidate genes, feature genes were further selected. The LASSO model (log(*λ*min) = −3.9958) retained six genes (Figure 3A): *ANXA3*, *MCEMP1*, *PRRG4*, *ANKRD22*, *VSIG4*, and *C8orf88*. RF analysis also identified six genes (Figures 3B,C): MCEMP1, PRRG4, VSIG4, C8orf88, OLAH, and H4C4 (mean Gini index = 1.280529). SVM–RFE yielded eight genes (Figure 3D): *C8orf88*, *MCEMP1*, *H4C4*, *MTARC1*, *PRRG4*, *VSIG4*, *TLR5*, and *H3C4*. Subsequently, four overlapping genes (*MCEMP1*, *PRRG4*, *VSIG4*, and *C8orf88*) were identified from the intersection of the three algorithms and were therefore defined as feature genes. Moreover, expression validation demonstrated that *MCEMP1*, *PRRG4*, and *VSIG4* were consistently upregulated in the IS group across both datasets (*p* < 0.05) (Figures 3E,F), confirming their stability and reliability as biomarkers. Notably, *C8orf88* was absent from the validation dataset and was therefore excluded from the final analysis. These biomarkers may serve as potential diagnostic indicators and provide novel insights into the immune–inflammatory mechanisms underlying IS. The scatter plot showed that the log_2_FC changes of the three biomarkers (*MCEMP1*, *PRRG4*, and *VSIG4*) exhibited identical trends (all upregulated) (Figure 3G). The Bland–Altman plot showed a mean difference of 0.190 with a 95% confidence interval ranging from −0.015 to 0.396, confirming good cross-platform consistency (although VSIG4 exhibited significant magnitude variations but consistent directional trends) (Figure 3H). The three biomarkers showed significant positive correlations with HPSE and EXT1 expression in the GAG metabolic pathway but no significant association with CHST2, suggesting their potential involvement in IS pathology through HPSE/EXT1-mediated GAG metabolic regulation (Figures 3I,J).

### 3.4 Nomogram model analysis for IS

**Figure 4:**
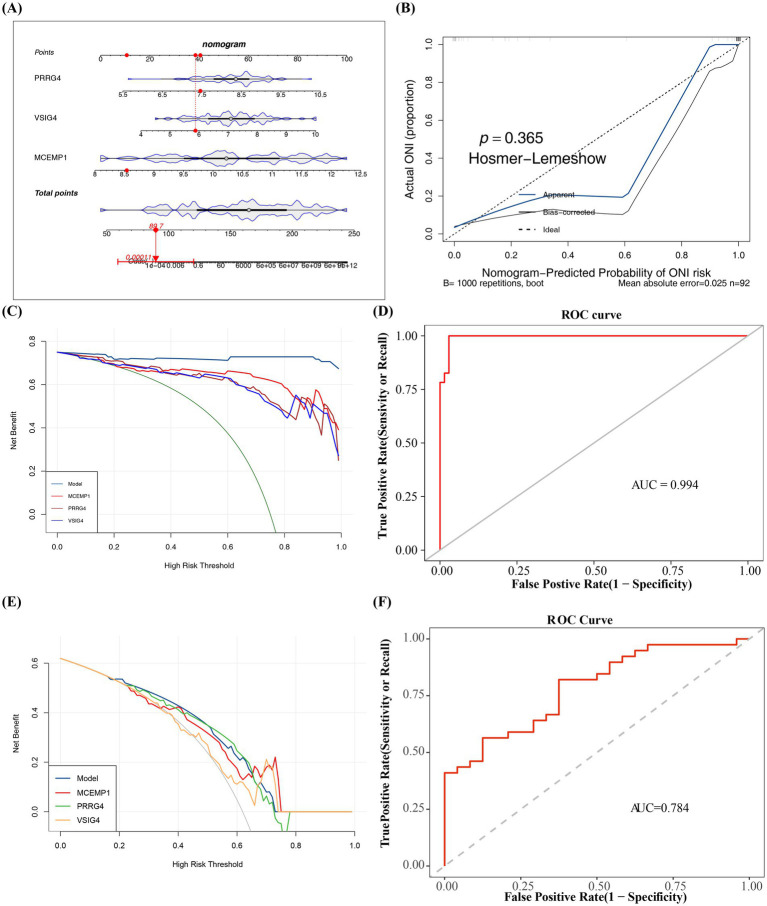
Construction and evaluation of the biomarker-based nomogram for ischemic stroke. **(A)** Nomogram integrating MCEMP1, PRRG4, and VSIG4. **(B)** Calibration curve for the nomogram. **(C)** Decision curve analysis of the nomogram and individual biomarkers. **(D)** ROC curve demonstrating the discriminative performance of the nomogram. **(E)** Decision curve analysis of the nomogram and individual biomarkers based on the external validation set (GSE16561). **(F)** ROC curve demonstrating the discriminative performance of the nomogram based on the external validation set (GSE16561).

The nomogram integrating the three biomarkers (MCEMP1, PRRG4, and VSIG4) was successfully constructed and visualized (Figure 4A). The calibration curve demonstrated good consistency between the predicted and observed probabilities of IS (*p* = 0.365), with the calibration slope approaching 1 (Figure 4B), indicating favorable predictive reliability. DCA revealed that the nomogram provided greater net clinical benefit across a wide threshold probability range compared with the biomarkers, “treat-all,” and “treat-none” strategies (Figure 4C), confirming its clinical utility. Moreover, ROC analysis showed remarkably high discriminatory capability, with an AUC of 0.994 (Figure 4D), supporting the robustness of the predictive model for differentiating IS patients from controls. For the external validation set (GSE16561), DCA demonstrated that the net benefit of the combined model was comparable to that of the single-gene models, with no significant advantage (Figure 4E). The ROC curve of the combined model had an AUC of 0.784 (Figure 4F), indicating that its diagnostic performance is acceptable, although its clinical utility remains limited. Collectively, these findings suggest that the nomogram based on MCEMP1, PRRG4, and VSIG4 offers a reliable and interpretable tool for predicting IS risk, with potential for aiding clinical decision-making and individualized diagnosis.

### 3.5 Enrichment pathway of biomarkers

**Figure 5:**
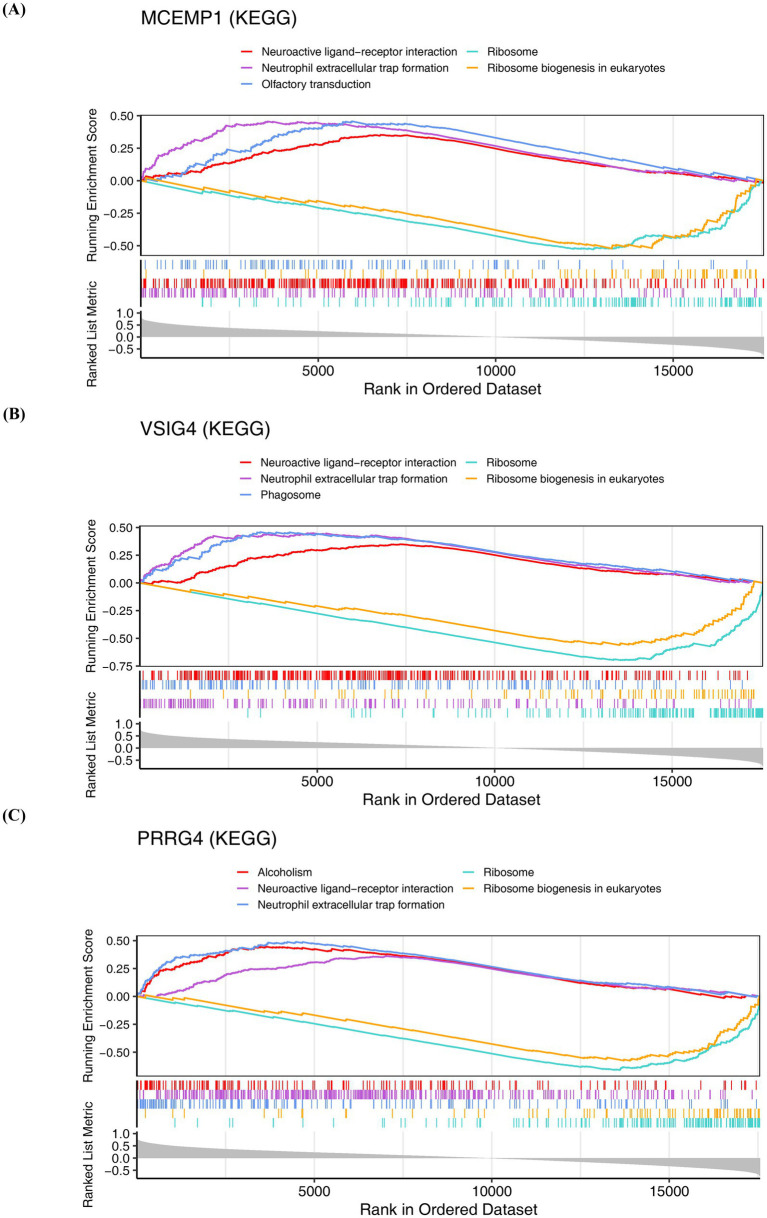
GSEA of the identified biomarkers in ischemic stroke. **(A–C)** Enriched pathways associated with MCEMP1, VSIG4, and PRRG4, respectively. The results highlight shared enrichment in ribosome-related pathways, neutrophil extracellular trap formation, and neuroactive ligand–receptor interaction.

GSEA identified a total of 134, 158, and 151 significantly enriched pathways for MCEMP1, VSIG4, and PRRG4, respectively (*p* < 0.05, |NES| > 1, FDR < 0.25) (Figures 5A−C; Supplementary Table 3). Notably, the top five enriched pathways for all three biomarkers consistently included the ribosome, neutrophil extracellular trap formation, ribosome biogenesis in eukaryotes, and neuroactive ligand–receptor interaction, highlighting their shared involvement in innate immune activation and translational regulation during IS. Moreover, MCEMP1 was uniquely enriched in olfactory transduction, while VSIG4 was associated with complement and phagocytic regulation, reflecting gene-specific regulatory patterns.

### 3.6 Immune infiltration in IS

**Figure 6:**
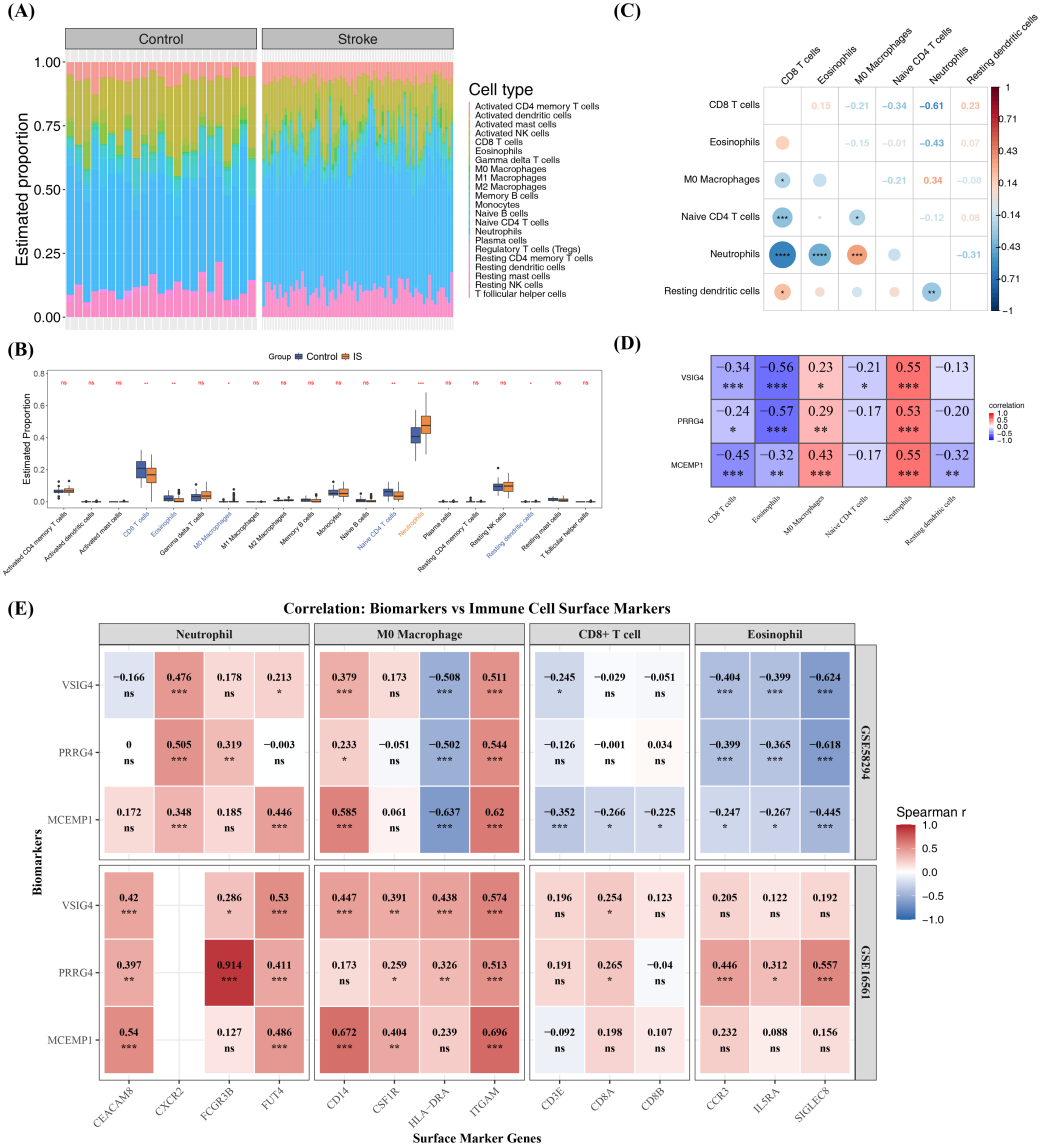
Immune infiltration characteristics in ischemic stroke and their associations with biomarkers. **(A)** Stacked bar plot of 22 immune cell populations. **(B)** Differential immune cell infiltration between the IS and control groups. **(C)** Correlation matrix among significantly altered immune cells. **(D)** Correlations between MCEMP1, PRRG4, VSIG4, and differential immune cells. ns represents insignificant, ^*^p < 0.05, ^**^p < 0.01, ^***^p < 0.001, ****p < 0.0001. **(E)** Correlation heatmap between biomarkers and immune cell surface marker genes in training and validation datasets.

CIBERSORT-based quantification and visualization of 22 immune cell types in the IS and control groups demonstrated that neutrophils constituted the dominant immune population in both groups (Figure 6A). Six immune cell types exhibited significant differential infiltration between the IS and control groups (*p* < 0.05): CD8 T cells, eosinophils, M0 macrophages, naive CD4^+^ T cells, neutrophils, and resting dendritic cells (Figure 6B). Spearman correlation analysis revealed that neutrophils were significantly negatively correlated with CD8^+^ T cells (cor = −0.61, *p* < 0.0001), eosinophils (cor = −0.43, *p* < 0.0001), and resting dendritic cells (cor = −0.31, *p* < 0.01), while positively correlated with M0 macrophages (cor = 0.34, *p* < 0.001). CD8 T cells were negatively correlated with naive CD4^+^ T cells (cor = −0.34, *p* < 0.001) (Figure 6C).

Further analysis linking biomarkers to differential immune cell infiltration revealed distinct association patterns (Figure 6D). MCEMP1 expression was positively correlated with neutrophils (cor = 0.55, *p* < 0.001) and M0 macrophages (cor = 0.43, *p* < 0.001) and negatively correlated with CD8 T cells (cor = −0.45, *p* < 0.001), eosinophils (cor = −0.32, *p* < 0.01), and resting dendritic cells (cor = −0.317, *p* < 0.01). PRRG4 was positively correlated with neutrophils (cor = 0.53, *p* < 0.001) and negatively correlated with eosinophils (cor = −0.57, *p* < 0.001). Association analysis between biomarkers and immune cell surface markers showed that in the training set (GSE58294), MCEMP1, PRRG4, and VSIG4 were positively correlated with neutrophil markers (FUT4 and CXCR2) and M0 macrophage markers (CD14 and ITGAM; MCEMP1 exhibited the strongest correlation with ITGAM, *r* = 0.620, *p* < 0.001), and negatively correlated with eosinophil markers (CCR3) and CD8^+^ T cell markers (*r* = −0.225 to −0.352, *p* < 0.05). In the validation set (GSE16561), the positive correlations with neutrophil and M0 macrophage markers remained significant, while the associations with eosinophil markers shifted direction (with PRRG4 showing positive correlations), and no significant associations were observed with CD8^+^ T cell markers (Figure 6E). VSIG4 showed a positive correlation with neutrophils (cor = 0.55, *p* < 0.001) and negative correlations with CD8 T cells (cor = −0.34, *p* < 0.001) and eosinophils (cor = −0.563, *p* < 0.001). These findings collectively indicate that the three biomarkers are intimately associated with neutrophil-driven inflammatory activation and diminished eosinophil/T cell immunity in IS.

### 3.7 Genomic and subcellular localization characteristics of biomarkers

**Figure 7:**
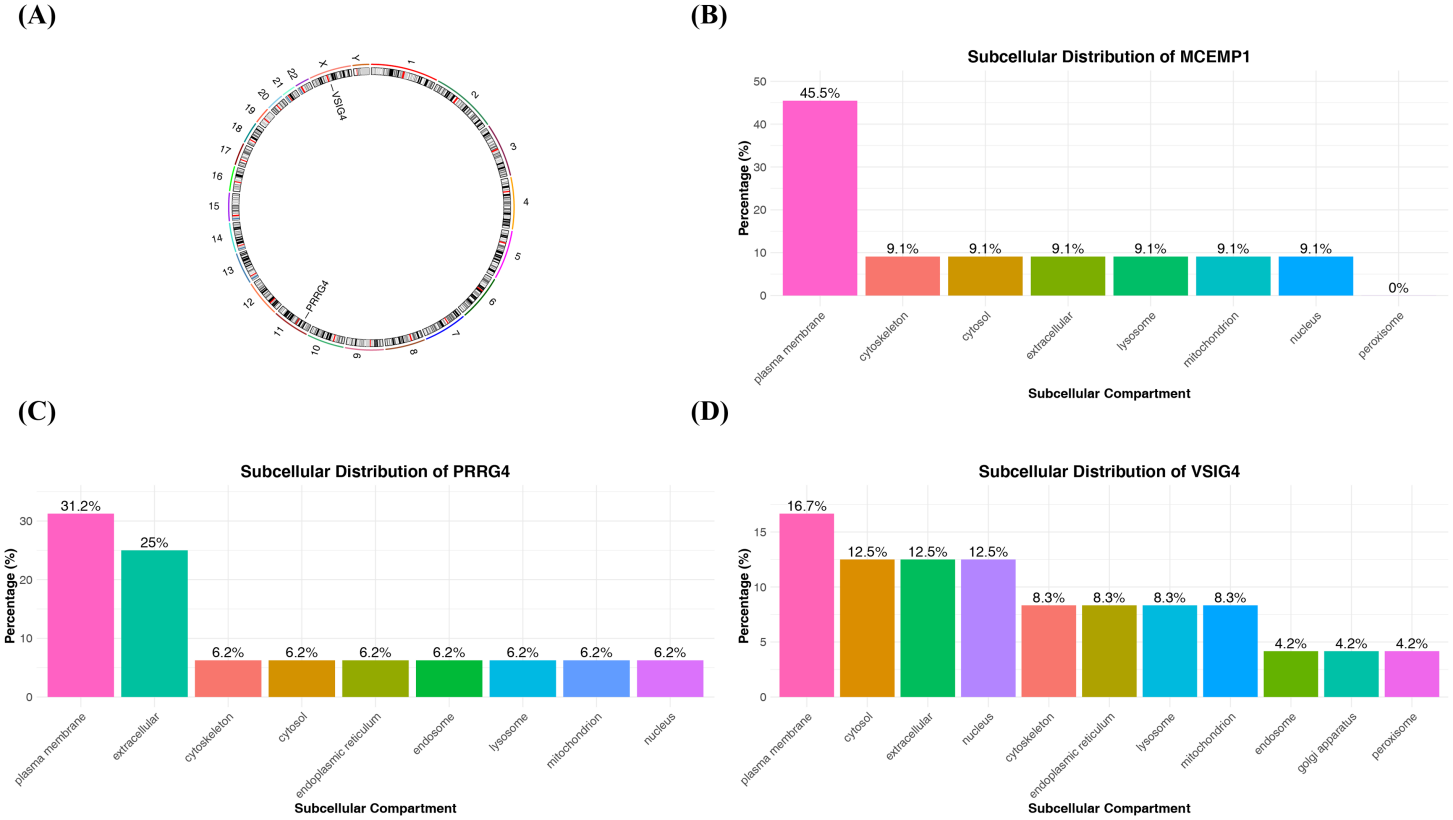
Genomic and subcellular localization of ischemic stroke biomarkers. **(A)** Chromosomal distribution of MCEMP1, PRRG4, and VSIG4. **(B–D)** Predicted subcellular localization of MCEMP1, PRRG4, and VSIG4, respectively.

Chromosomal mapping showed that the biomarkers were located on distinct genomic regions, with PRRG4 positioned on chromosome 11 and VSIG4 located on the X chromosome, while no valid chromosomal annotation was found for MCEMP1 in the reference database (Figure 7A). The spatial separation of these genes suggests that they are not physically clustered within a single chromosomal locus.

Subcellular localization prediction further revealed that all three biomarkers (MCEMP1, PRRG4, and VSIG4) were primarily localized to the plasma membrane (Figures 7B−D), implying their potential roles in mediating cell–cell communication and immune receptor signaling in IS.

### 3.8 Network-based functional and therapeutic relevance of biomarkers

**Figure 8:**
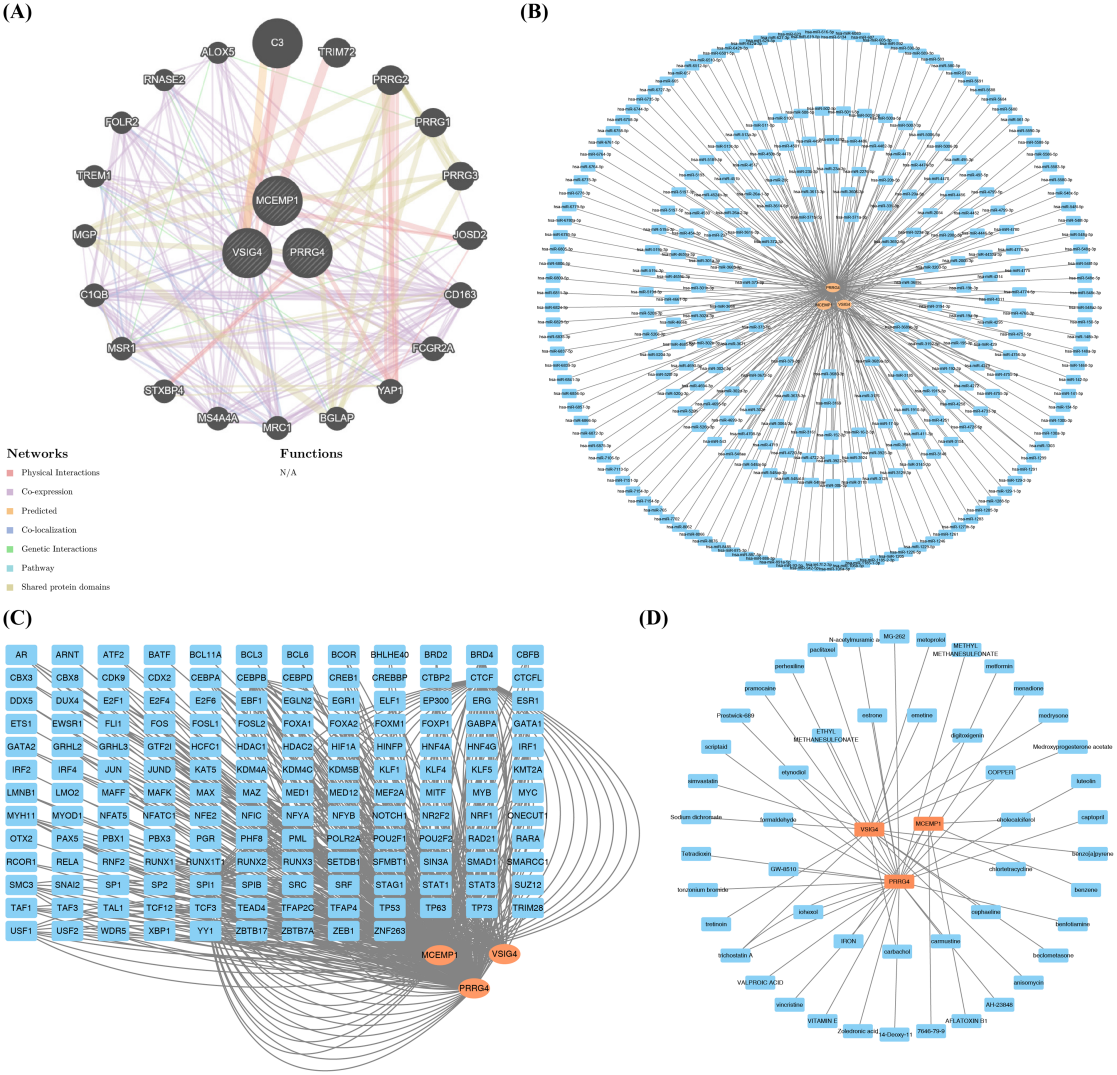
Network-based analysis of the functional regulation and therapeutic relevance of biomarkers. **(A)** GeneMANIA network of MCEMP1, PRRG4, and VSIG4. **(B)** miRNA–mRNA regulatory network. **(C)** TF–mRNA regulatory network. **(D)** Drug–gene interaction network highlighting candidate compounds, including trichostatin A.

GeneMANIA revealed that the three biomarkers were embedded in a dense functional interaction network, particularly co-expressed with immune-related genes (Figure 8A). For instance, MCEMP1 and VSIG4 were closely associated with TREM1 and MS4A6A, implying a cooperative role in inflammatory activation and immune regulation. PRRG4 formed a structural domain–based interaction cluster with PRRG2 and PRRG3, suggesting potentially conserved protein functions. Additionally, the complement component C3 was linked to multiple interaction axes, highlighting its immune regulatory significance.

Upstream regulatory analysis identified 55, 201, and 21 miRNAs targeting VSIG4, PRRG4, and MCEMP1, respectively (Figure 8B). Among them, several miRNAs, such as hsa-miR-5006-3p, hsa-miR-4755-5p, and hsa-miR-4446-5p, were predicted to regulate more than one biomarker, indicating convergent post-transcriptional control. Similarly, TF analysis identified 62 TFs for VSIG4, 221 for PRRG4, and 236 for MCEMP1 (Figure 8C), with key regulators, including CTCF, SPI1, and CEBPB, further supporting their involvement in immune–inflammatory signaling networks.

In addition, drug–gene interaction networks predicted 51 biomarker–compound interactions, including 5 targeting MCEMP1, 17 targeting VSIG4, and 29 targeting PRRG4 (Figure 8D). Notably, trichostatin A (TSA) was identified as a shared interacting compound for all three biomarkers, suggesting promising therapeutic potential for modulating coordinated immune dysregulation in IS.

### 3.9 Clinical trial validation of biomarkers

**Figure 9:**
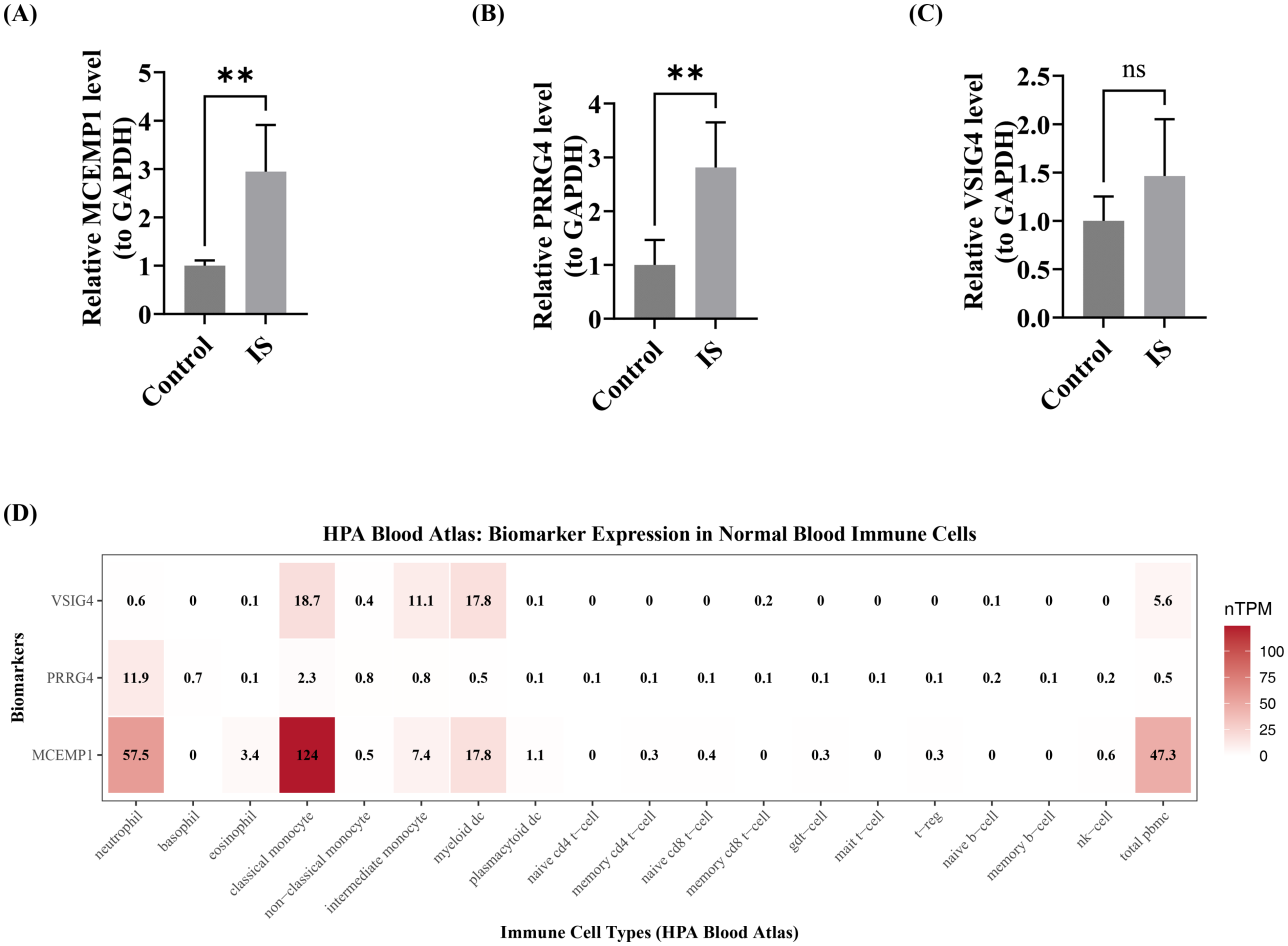
Clinical RT-qPCR validation of biomarker expression in ischemic stroke. **(A–C)** Relative expression levels of MCEMP1, PRRG4, and VSIG4 in blood samples from IS patients and healthy controls; ns represents insignificant, ^*^p < 0.05, ^**^p < 0.01. **(D)** Heatmap of biomarker expression abundance across circulating immune cell subsets (nTPM) in the Human Protein Atlas Blood Atlas.

Relative to the control group, expression levels of MCEMP1 and PRRG4 were significantly elevated in IS patients (*p* < 0.05), whereas VSIG4 showed an upward expression tendency that failed to achieve statistical significance (*p* > 0.05) (Figures 9A–C). The observed effect size for VSIG4 was *d* = 1.026, with a current statistical power of only 0.299, indicating a risk of false negatives and insufficient sample size to provide robust statistical evidence. Collectively, these observations indicate that MCEMP1 and PRRG4 may serve key functions in IS pathogenesis, while the contributory role of VSIG4 remains to be clarified in future studies.

### 3.10 Analysis of expression specificity and abundance of biomarkers in circulating immune cell subsets

The results showed that MCEMP1 had the highest expression abundance in classical monocytes (nTPM = 124), followed by neutrophils (57.5) and total peripheral blood mononuclear cells (PBMCs) (47.3), with moderate expression in myeloid dendritic cells (17.8) and intermediate monocytes (7.4), while it was scarcely expressed in lymphocyte subsets such as T cells, B cells, and NK cells (nTPM ≈ 0–0.4). PRRG4 was specifically concentrated in neutrophils (11.9), and expression levels in other immune cell subsets were all low (nTPM ≤ 2.3). VSIG4 was highly expressed mainly in classical monocytes (18.7), myeloid dendritic cells (17.8), and intermediate monocytes (11.1), with an expression level of 5.6 in total PBMCs and only 0.6 in neutrophils and was hardly detected in lymphocytes. Collectively, the three biomarkers presented a distinct myeloid-biased expression profile in circulating immune cells: MCEMP1 was mainly in monocytes/neutrophils, PRRG4 in neutrophils, and VSIG4 in monocytes/dendritic cells, with none exhibiting lymphocyte-specific expression (Figure 9D).

## 4 Discussion

The IS acute phase is characterized by immune–inflammatory dysregulation and BBB dysfunction induced by ischemia–reperfusion, making identification of upstream regulatory hub molecules and validation of actionable biomarkers an urgent clinical challenge (1, 2). As core components of the cerebral endothelial glycocalyx and ECM, GAGs are deeply involved in post-stroke inflammatory amplification and repair initiation by regulating barrier permeability, complement assembly, and immune cell–endothelium interaction (8, 9). In this study, 30 GAG-related candidate genes were initially obtained through integrated differential expression analysis and WGCNA, from which three hub genes (*MCEMP1*, *PRRG4*, and *VSIG4*) were ultimately established as core diagnostic biomarkers for IS. All three genes demonstrated marked upregulation in patient specimens and were functionally enriched in immune–inflammatory pathways, including the ribosomal pathway, neutrophil extracellular trap formation, and neuroactive ligand–receptor interactions. Immune infiltration profiling demonstrated strong positive correlations of these genes with neutrophils and M0 macrophages, alongside negative correlations with CD8^+^ T cells and eosinophils. A predictive nomogram incorporating the three hub genes was subsequently developed, yielding an AUC of 0.994 and demonstrating superior clinical discriminatory performance. Furthermore, GSEA and upstream regulatory network inference highlighted the pivotal involvement of these genes in inflammatory and metabolic regulation, and TSA was proposed as a candidate compound for potential targeted therapeutic interventions.

MCEMP1 is upregulated in various inflammatory diseases as well as cardio-cerebrovascular diseases, and its inhibition can alleviate inflammatory injury, being closely associated with innate immunity activation (33). Notably, damage to the endothelial glycocalyx with GAGs as the core component can enhance leukocyte adhesion, complement assembly, and microcirculatory disturbance, thereby promoting cerebrovascular disease progression. The core mechanism lies in “membrane microenvironment” shaping for myeloid cell adhesion and activation (33, 34). In the IS acute phase, the mobilization level of myeloid cells is directly correlated with patient prognosis, and the neutrophil-to-lymphocyte ratio has been validated as a prognostic biomarker. The present study found that MCEMP1 was significantly upregulated in the peripheral blood of IS patients and was positively correlated with the infiltration of neutrophils and M0 macrophages while negatively correlated with the infiltration of CD8^+^ T cells. This expression pattern is highly consistent with the overall immune characteristic of predominant innate immunity and suppressed adaptive immunity in the IS acute phase. Combined with existing evidence that GAG glycocalyx damage promotes myeloid adhesion and activation, we speculated that MCEMP1 may serve as a detectable peripheral marker on the regulatory axis of “abnormal GAG metabolism–myeloid inflammatory amplification,” providing a potential target for the accurate assessment of early inflammatory status in IS (35–37). Clinical validation further confirmed that MCEMP1 was significantly upregulated in IS patients compared with controls (*p* < 0.05), supporting its reliability as a peripheral biomarker.

Proline-rich and Gla domain 4 (PRRG4) belongs to the membrane protein family containing the *γ*-carboxyglutamic acid (Gla) domain, with the core function of regulating membrane signal transduction and intercellular interaction. The latest conditional knockout study in mice confirmed that PRRG4 deficiency can lead to abnormal central excitability and behavioral changes, suggesting its key role in the regulation of brain functional homeostasis (38). The present study confirmed that PRRG4 was significantly upregulated in the peripheral blood of IS patients and was significantly correlated with the infiltration characteristics of immune cells, such as neutrophils and eosinophils. Combined with its plasma membrane localization and central homeostatic regulatory function, as verified by recent animal experiments, PRRG4 is hypothesized to be involved in IS pathology through plasma membrane surface signal transduction. Its upregulation in peripheral blood and correlation with myeloid cells suggest that PRRG4 participates in signal remodeling at the interface between peripheral immune cells and endothelial cells; however, experimental investigations have yet to validate its specific mechanism of action. The significant upregulation of PRRG4 in clinical IS samples (*p* < 0.05) provides preliminary experimental support for its pathological involvement, though further mechanistic studies are warranted.

V-set and immunoglobulin domain-containing 4 (VSIG4), also known as the complement receptor of the immunoglobulin superfamily (CRIg), is a specific complement receptor on the surface of tissue-resident macrophages. It participates in the regulation of immune homeostasis by negatively regulating T cell activation, modulating phagocytic clearance function, and inflammatory metabolic programs (39). Recent key mechanistic studies have shown that VSIG4 can directly bind to heparan sulfate (HS), a core subtype of GAGs, and HS and complement components C3b/iC3b compete for binding sites on VSIG4, suggesting that VSIG4 occupies a unique nodal position in the GAG-complement cross-regulatory network (40). Excessive activation of the complement system during the IS acute phase is closely related to vascular injury and immune disorder, and GAGs such as HS can affect the signal transduction of the complement-phagocytosis axis by regulating the binding efficiency of VSIG4 to C3b/iC3b, which provides direct molecular evidence for “abnormal GAG metabolism regulating the complement-macrophage pathway” (41). The present study found that VSIG4 was significantly upregulated in the peripheral blood of IS patients and was significantly correlated with increased neutrophil infiltration and decreased CD8^+^ T cell infiltration. This expression signature is highly consistent with its biological functions: VSIG4 exerts a negative regulatory effect on T cell activation, which corresponds to its negative correlation with CD8^+^ T cells; its expression on tissue-resident macrophages and complement regulatory function form a synergistic effect with neutrophil-dominated innate immune activation.

Expression specificity further confirms that MCEMP1, PRRG4, and VSIG4 exhibit a distinct myeloid-biased expression profile in circulating immune cell subsets, with VSIG4 being predominantly highly expressed in classical monocytes and myeloid dendritic cells. This expression restriction may account for the failure to detect intergroup differences in total peripheral blood RNA, thereby supporting its reliability as a peripheral biomarker.

GSEA analysis revealed significant enrichment of ribosome/ribosome biogenesis, neutrophil extracellular trap (NET) formation, and neuroactive ligand–receptor interaction pathways, consistent with the molecular landscape of systemic stress response and innate immune activation in the IS acute phase. Ribosome and ribosome biogenesis pathway activation indicates protein translation reprogramming under stress conditions, and upregulation of the ribosome biogenesis gene cluster in IS has been confirmed to be associated with inflammatory cell infiltration (42). NET formation pathway enrichment is closely linked to thrombosis, reperfusion injury, and peripheral–central immune imbalance; NET-related markers can effectively assess stroke severity and prognosis, and targeted NET intervention has shown neuroprotective effects in animal models (43). This is consistent with the increased neutrophil proportion and the correlation of MCEMP1/VSIG4 with myeloid cells observed in this study, confirming that NETs represent an important peripheral immune amplification phenotype in IS. Enrichment of the neuroactive ligand–receptor interaction pathway further suggests involvement of the neuroimmune-endocrine axis, consistent with transcriptomic evidence of dynamic disturbance after cerebral ischemia (44, 45).

Bioinformatics-based biomarker-specific pathway analysis revealed that MCEMP1-enriched pathways concentrated in innate immune and inflammatory regulation, consistent with its transmembrane signal amplification function in myeloid cells; against the background of GAG glycocalyx damage, enhanced receptor aggregation may further amplify MCEMP1-dependent membrane signaling, echoing the “inflammation-complement-endothelium axis” regulatory network in IS (46). VSIG4-enriched pathways concentrated in complement and phagocytosis regulation, consistent with their core function as complement receptors, and the VSIG4-HS-C3b/iC3b competitive binding mechanism establishes a direct molecular link between abnormal GAG metabolism and complement-macrophage pathway dysregulation. PRRG4-enriched pathways involve membrane signal transduction and metabolic responses. Together with its plasma membrane localization and central homeostatic regulatory function, these findings suggest that PRRG4 participates in signal remodeling in IS.

Machine-based predictions indicate that all three biomarkers are significantly associated with ribosome biogenesis, NET formation, and neuroactive ligand–receptor interaction pathways, collectively forming the core molecular network underlying innate immune activation and inflammatory amplification in the IS acute phase.

Immune infiltration analysis indicated that the peripheral immune microenvironment in IS is predominantly shaped by neutrophil accumulation, which exhibits inverse correlations with CD8^+^ T cells, eosinophils, and resting dendritic cells, while showing a positive association with M0 macrophages. This immune configuration reflects an acute-phase pathological state characterized by concurrent innate immune hyperactivation and adaptive immune suppression, both of which are closely linked to stroke severity and clinical outcomes (43). Biomarker correlation profiling further revealed that MCEMP1 expression was positively associated with neutrophils and M0 macrophages yet inversely associated with CD8^+^ T cells, a pattern consonant with its function as an SCF-KIT signaling adaptor that promotes myeloid-driven inflammatory cascades. Given its identity as a GAG metabolism–related biomarker, MCEMP1 implies that dysregulated GAG metabolism may contribute to myeloid cell-dependent inflammatory amplification through remodeling of the plasma membrane microenvironment (36), echoing evidence that endothelial glycocalyx damage promotes myeloid cell adhesion and activation (47). VSIG4 exhibited a similar correlation profile, consistent with its function as a complement receptor on tissue-resident macrophages that negatively regulates T cell activation (48). Because immune infiltration analysis is limited by CIBERSORT’s bulk-tissue optimization (introducing bias in acute stroke peripheral blood), unexcluded confounders (infection/stress/medication), and lack of flow cytometry validation, inferences such as eosinophil-key gene links are computational predictions/biological hypotheses requiring experimental confirmation.

As a key node in the GAG-related gene module, the upregulation of VSIG4 may reflect the reprogramming of the complement-phagocytosis regulatory axis in the IS acute phase against the backdrop of GAG metabolic disorder, and its expression alteration in peripheral blood may indicate the activation of the complement-macrophage pathway induced by GAG network imbalance (49) PRRG4 was significantly correlated with neutrophils and eosinophils, and its plasma membrane localization characteristic was consistent with the functional mode of the Gla domain-containing protein family. The functional exertion of Gla domain-containing proteins is often dependent on the synergistic interaction with ECM components, including GAGs (50). As a member of the GAG-related gene network, the upregulation of PRRG4 and its association with granulocyte infiltration suggest that altered GAG metabolism impairs the assembly of Gla protein-mediated plasma membrane signaling platforms. Recent studies have confirmed the role of PRRG4 in the regulation of central functional homeostasis (51); combined with its plasma membrane localization and immune correlation characteristics, this molecule may play a bridging role in peripheral–central immune signal transduction.

Based on the results of the drug–gene interaction network analysis, this study identified potential therapeutic targets for the precision treatment of IS. A total of 51 biomarker–compound interactions were predicted in the present study, among which TSA exhibited unique therapeutic value as the only compound that simultaneously targets three GAGs-related genes, namely *MCEMP1*, *VSIG4*, and *PRRG4* (52). As a histone deacetylase inhibitor, TSA has been proven to exert neuroprotective effects in various neurological disorders. Considering that all three biomarkers are involved in the regulation of immune inflammation, TSA may synergistically modulate GAG-mediated inflammatory responses through epigenetic modification pathways, thereby ameliorating the immune dysregulation status post-IS (53). In addition, the 17 compounds targeting VSIG4 and 29 compounds targeting PRRG4 provide a candidate molecular library for a multi-target therapeutic strategy. As an essential component of the ECM, GAGs play a pivotal role in BBB disruption and inflammatory infiltration after IS (54); therefore, pharmacological intervention targeting GAG-related genes may yield dual benefits: regulating the inflammatory microenvironment while preserving matrix homeostasis. However, the drug prediction in this study was based on bioinformatics analysis, and its actual efficacy and safety remain to be verified by *in vitro* cellular experiments and animal models. Future studies should focus on the regulatory effects of TSA on these three target genes and the underlying mechanisms of their downstream signaling pathways in IS models.

In summary, distinct from previous studies that focus primarily on conventional inflammatory or vascular pathways, this study identified IS-related biomarkers from the perspective of GAG metabolism dysregulation. By integrating differential expression analysis, WGCNA, and multiple machine-learning algorithms, we successfully screened three core biomarkers (MCEMP1, PRRG4, and VSIG4) from peripheral blood transcriptome data and constructed a high-performance nomogram prediction model. Functional enrichment revealed their involvement in ribosome biogenesis and NET formation, while immune infiltration analysis demonstrated neutrophil-dominated characteristics. Notably, drug prediction identified TSA as a potential multi-target therapeutic agent, providing novel insights into the interplay between GAG metabolism and immune dysregulation in IS pathogenesis.

However, several limitations warrant consideration. First, the relatively small sample size from the GEO platform and the lack of detailed clinical stratification may affect generalizability. Second, findings regarding immune infiltration, pathway enrichment, and drug prediction require validation through independent clinical cohorts and experimental models. Furthermore, current clinical validation is limited by a small sample size; it lacks statistical power to confirm biomarker associations with stroke severity, prognosis, or clinical characteristics, nor does it adjust for potential confounders (age, sex, and vascular risks). Future studies should cover diverse stroke subtypes, severities, and prognoses using multivariate regression to establish clinical translational value. Third, the LM22 signature in CIBERSORT was optimized for PBMCs; high-abundance granulocyte/platelet RNA in whole blood may skew deconvolution accuracy, especially for neutrophil/macrophage estimates. Though the Z-score normalized, it could not fully eliminate whole blood sample biases. Future studies should validate these biomarkers in multicenter prospective cohorts by incorporating GAG fragment detection and elucidating their regulatory mechanisms across different IS stages using single-cell sequencing and functional experiments.

## Data Availability

The original contributions presented in the study are included in the article/Supplementary material, further inquiries can be directed to the corresponding author.

## Footnotes

1
https://www.genecards.org/

2
https://genemania.org/

3
https://www.targetscan.org/vert_80/

4
https://mirdb.org/

5
https://guolab.wchscu.cn/hTFtarget/#!/

6
http://dsigdb.tanlab.org/

7
https://www.graphpad.com/

## Supplementary material

SUPPLEMENTARY TABLE S1 127 GAG metabolism-related genes and baseline clinical data used in RT-qPCR analysis.

SUPPLEMENTARY TABLE S2 GO and KEGG enrichment analysis results.

SUPPLEMENTARY TABLE S3 Enrichment pathways of biomarkers.
